# Supplementary material for: A multi-site German validation of the Interoceptive Accuracy Scale and its relation to psychopathological symptom burden
Source: Commun Psychol. Author manuscript; Available in PMC 2024 Aug 29. (PMC11332230; doi:10.1038/s44271-023-00016-x)
Supplement: Supplementary material [file EMS198321-supplement-Supplementary_material.pdf]

Supplementary Information

Supplementary Notes 1: Summary of all differences between German IAS versions in relation to the final one (Mainz Version, see Supplementary Notes 2)

Potsdam version

The only difference between the Potsdam and the final IAS version was the usage of a formal formulation of item 5 (*Harndrang verspüren* instead of *Wasser lassen*).

Vienna version

The Vienna version differed from the final IAS version in the initial item wording which was *akkurat wahrnehmen* instead of *genau wahrnehmen*. Furthermore, item 5 was, as for the Potsdam version, formulated formally.

Giessen version

The initial wording in the Giessen version of the scale was *Ich kann genau wahrnehmen* which differed from *Ich kann immer genau wahrnehmen* from the final IAS version. In addition, the wording of the 5-point Likert Scale range from *fast nie* to *fast immer* rather than S*tarke Ablehnung* to *Starke Zustimmung*.

Supplementary Notes 2: The final German version of the Interoceptive Accuracy Scale (IAS; Mainz Version)

Nachfolgend finden Sie einige Aussagen darüber, wie genau Sie bestimmte Körperempfindungen wahrnehmen können. Bitte bewerten Sie auf der Skala, was Sie glauben, wie gut Sie jedes einzelne Körpersignal wahrnehmen können. Wenn Sie zum Beispiel das Gefühl haben, Wasser lassen zu müssen und dann auf der Toilette allerdings merken, dass Sie nicht müssen, würden Sie Ihre Genauigkeit, das Körpersignal wahrzunehmen, niedrig einschätzen.

Bitte bewerten Sie, wie gut Sie diese Signale ohne externe Hinweise wahrnehmen können. Zum Beispiel, wenn Sie nur wahrnehmen können, wie schnell Ihr Herz schlägt, wenn Sie Ihren Puls fühlen, würde dies nicht als genaue Wahrnehmung innerer Zustände zählen

1. Ich kann immer genau wahrnehmen, wenn mein Herz schnell schlägt.
2. Ich kann immer genau wahrnehmen, wenn ich Hunger habe.
3. Ich kann immer genau wahrnehmen, wenn ich schnell atme.
4. Ich kann immer genau wahrnehmen, wenn ich durstig bin.
5. Ich kann immer genau wahrnehmen, wenn ich Wasser lassen muss.
6. Ich kann immer genau wahrnehmen, wenn ich einen Drang zum Stuhlgang habe.
7. Ich kann immer genau wahrnehmen, wenn ich auf verschiedene Geschmacksrichtungen stoße.
8. Ich kann immer genau wahrnehmen, wenn ich erbrechen werde.
9. Ich kann immer genau wahrnehmen, wenn ich niesen werde.
10. Ich kann immer genau wahrnehmen, wenn ich husten werde.
11. Ich kann immer genau wahrnehmen, wenn mir heiß/kalt ist.
12. Ich kann immer genau wahrnehmen, wenn ich sexuell erregt bin.
13. Ich kann immer genau wahrnehmen, wenn ich Blähungen haben werde.
14. Ich kann immer genau wahrnehmen, wenn ich aufstoßen werde.
15. Ich kann immer genau wahrnehmen, wenn meine Muskeln müde sind bzw. ich Muskelkater habe.
16. Ich kann immer genau wahrnehmen, wenn ich einen blauen Fleck bekomme.
17. Ich kann immer genau wahrnehmen, wenn ich Schmerzen habe.
18. Ich kann immer genau wahrnehmen, wenn mein Blutzucker niedrig ist.
19. Ich kann immer genau wahrnehmen, wenn mich jemand eher liebevoll statt nicht liebevoll berührt.
20. Ich kann immer genau wahrnehmen, wenn etwas kitzlig ist.
21. Ich kann immer genau wahrnehmen, wenn etwas juckt.

Skala. Starke Ablehnung; Ablehnung; Weder Zustimmung noch Ablehnung; Zustimmung; Starke Zustimmung.

Supplementary Notes 3

Supplementary Table 1

Descriptives of German versions of the IAS at the Item level

Item	*M*					*SD*					$r_{it(i)}$					P_i					Skewness					Kurtosis				
No.	*M1*	*M2*	*Po*	*Vi*	*Gi*	*M1*	*M2*	*Po*	*Vi*	*Gi*	*M1*	*M2*	*Po*	*Vi*	*Gi*	*M1*	*M2*	*Po*	*Vi*	*Gi*	*M1*	*M2*	*Po*	*Vi*	*Gi*	*M1*	*M2*	*Po*	*Vi*	*Gi*
1	3.8	3.8	3.7	3.6	4.1	0.9	0.9	1.1	1.0	0.9	.43	.38	.35	.35	.26	.71	.71	.74	.73	.81	-0.48	-0.78	-0.79	-0.73	-0.99	-0.58	0.58	-0.10	-0.13	1.11
2	3.5	3.9	3.9	3.8	4.3	1.0	1.0	1.0	1.0	0.9	.37	.42	.40	.34	.32	.63	.72	.78	.76	.85	-0.35	-0.74	-0.81	-0.72	-1.43	-0.71	-0.04	-0.09	-0.34	1.88
3	3.9	3.9	3.8	3.8	4.2	0.9	0.9	1.0	1.0	1.0	.45	.47	.42	.43	.39	.72	.71	.77	.77	.83	-0.50	-0.74	-0.82	-0.78	-1.03	-0.31	0.47	0.09	0.14	0.52
4	3.6	3.9	3.9	3.9	4.0	1.1	1.1	1.1	1.1	1.1	.31	.37	.38	.37	.38	.66	.71	.78	.77	.80	-0.50	-0.77	-0.84	-0.81	-0.99	-0.63	-0.16	-0.23	-0.19	-0.12
5	4.2	4.4	4.2	4.2	4.6	0.8	0.8	0.9	0.9	0.7	.38	.49	.46	.40	.39	.79	.84	.84	.84	.92	-1.00	-1.37	-1.19	-1.14	-1.98	0.89	2.17	1.01	1.02	3.87
6	4.2	4.4	4.3	4.2	4.6	0.8	0.8	0.8	0.8	0.7	.37	.52	.50	.43	.46	.80	.84	.85	.84	.92	-1.07	-1.37	-1.27	-1.01	-1.91	1.40	2.55	1.75	0.64	4.06
7	3.8	4.0	4.0	3.9	4.4	0.9	0.9	0.9	1.0	0.8	.47	.53	.47	.43	.45	.70	.76	.80	.78	.87	-0.58	-0.82	-0.84	-0.71	-1.13	-0.26	0.65	0.29	0.01	1.33
8	3.9	4.1	4.0	3.9	4.2	0.9	0.9	1.0	1.0	0.9	.32	.48	.43	.38	.51	.73	.77	.80	.77	.84	-0.59	-0.85	-0.89	-0.76	-1.06	-0.40	0.17	0.24	0.02	0.56
9	4.2	4.2	4.1	4.0	4.5	0.8	0.8	0.9	0.9	0.7	.44	.54	.51	.50	.44	.80	.81	.82	.80	.89	-0.89	-1.05	-0.91	-0.89	-1.10	0.60	1.32	0.54	0.54	0.76
10	3.9	4.1	3.9	3.8	4.3	0.9	0.9	1.0	1.0	0.8	.41	.60	.55	.53	.52	.74	.77	.78	.76	.85	-0.72	-0.79	-0.75	-0.61	-0.99	0.33	0.30	0.12	-0.31	0.68
11	4.2	4.2	4.1	4.1	4.6	0.8	0.8	0.9	0.9	0.7	.48	.54	.54	.49	.45	.79	.81	.83	.83	.91	-0.86	-1.12	-1.01	-0.98	-1.72	0.45	1.27	0.71	0.58	3.24
12	4.1	4.1	4.1	4.0	4.2	0.8	0.8	0.9	0.9	0.8	.42	.54	.49	.43	.46	.77	.78	.81	.80	.84	-0.76	-0.96	-1.01	-0.92	-1.12	0.50	0.94	0.78	0.48	1.64
13	3.5	3.8	3.9	3.8	3.9	1.0	1.0	0.9	0.9	0.9	.44	.57	.57	.53	.52	.62	.70	.78	.76	.78	-0.32	-0.59	-0.72	-0.68	-0.63	-0.65	-0.16	0.23	0.14	-0.14
14	3.5	3.7	3.6	3.6	3.8	1.0	1.0	1.1	1.0	1.0	.48	.60	.53	.55	.53	.62	.66	.72	.72	.76	-0.27	-0.40	-0.50	-0.53	-0.60	-0.65	-0.43	-0.48	-0.38	-0.11
15	4.1	4.1	4.0	3.9	4.4	0.7	0.8	0.9	1.0	0.8	.47	.57	.50	.40	.50	.76	.78	.79	.79	.88	-0.63	-1.03	-0.88	-0.83	-1.48	0.50	1.20	0.55	0.46	2.71
16	2.6	3.0	2.5	2.5	2.9	1.1	1.2	1.1	1.1	1.2	.43	.49	.40	.46	.48	.39	.49	.50	.50	.58	0.48	0.14	0.50	0.43	0.10	-0.39	-0.92	-0.49	-0.66	-0.82
17	4.1	4.1	4.1	4.0	4.4	0.8	0.8	0.9	0.9	0.7	.50	.58	.53	.50	.52	.77	.78	.82	.80	.88	-0.81	-0.94	-0.82	-1.00	-1.20	0.38	1.22	0.37	0.93	1.67
18	2.6	2.7	2.4	2.3	2.7	1.1	1.1	1.1	1.1	1.2	.41	.34	.28	.28	.41	.39	.44	.48	.46	.54	0.41	0.15	0.43	0.66	0.18	-0.65	-0.72	-0.77	-0.39	-0.95
19	4.1	4.2	4.0	3.9	4.2	0.8	0.8	1.0	1.0	0.9	.39	.59	.48	.40	.49	.78	.79	.80	.78	.84	-0.70	-0.94	-0.90	-0.84	-1.33	0.31	0.98	0.42	0.36	1.83
20	4.1	4.3	3.5	3.4	4.4	0.8	0.8	1.1	1.1	0.9	.35	.50	.48	.40	.48	.78	.81	.71	.68	.88	-0.87	-1.12	-0.46	-0.31	-1.72	0.78	1.42	-0.58	-0.61	3.08
21	4.2	4.4	3.4	3.3	4.5	0.7	0.7	1.1	1.1	0.6	.51	.57	.49	.41	.58	.81	.84	.68	.65	.91	-1.00	-1.05	-0.33	-0.28	-1.29	1.56	2.34	-0.62	-0.66	1.42

Note. *M* = Mean; *SD* = Standard Deviation; $r_{it(i)}$ = Item-Total Correlation; P_i = Item Difficulty; Scale from 1-5. M1 = Mainz Sample 1 (Confirmatory); M2 = Mainz Sample 2 (Exploratory); Vi = Vienna Version; Po = Potsdam Version; Gi = Giessen Version.

Supplementary Notes 4

Supplementary Figure 1

Age distribution across samples and versions

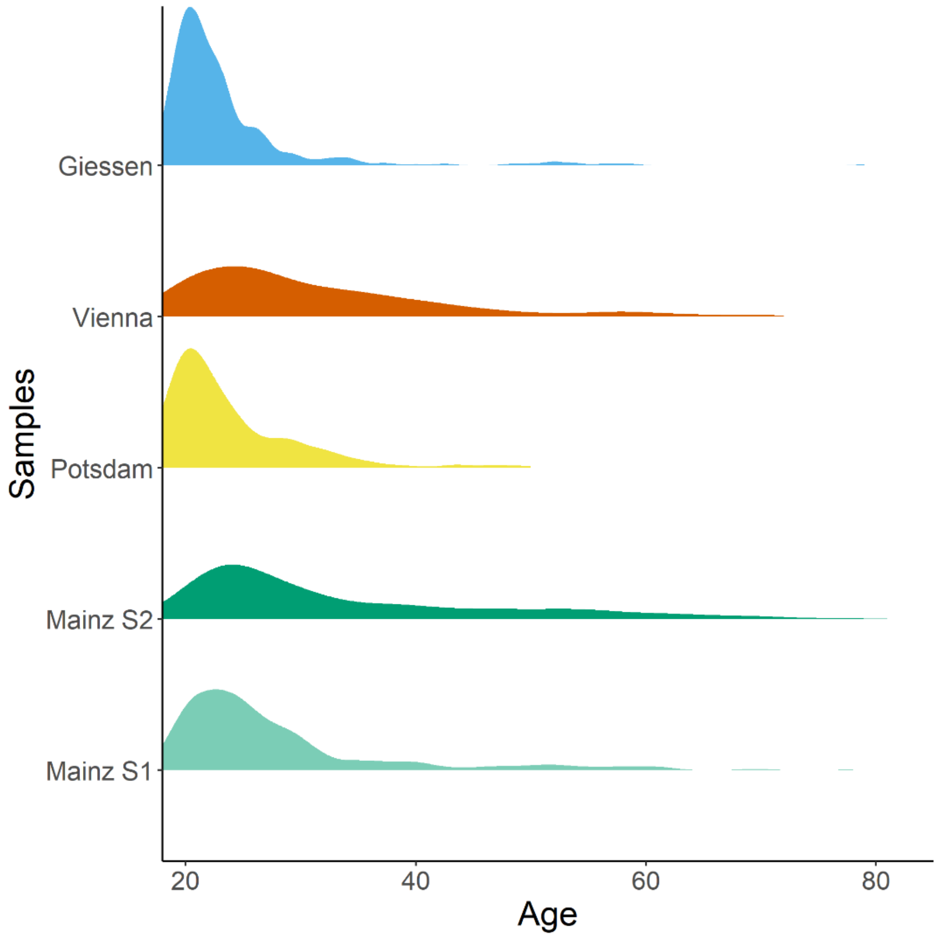

Supplementary Notes 5: Correlational analysis across IAS Versions

Supplementary Table 2

Correlational analysis across questionnaires for the Mainz version of the IAS

Pearson's *r*	*M* (*SD*)	1 [95 % *CI*]	2 [95 % *CI*]	3 [95 % *CI*]	4 [95 % *CI*]	5 [95 % *CI*]	6 [95 % *CI*]	7 [95 % *CI*]	8 [95 % *CI*]	9 [95 % *CI*]	10 [95 % *CI*]	11 [95 % *CI*]	12 [95 % *CI*]	13 [95 % *CI*]
1. IAS Mainz 1 (*N* = 484)	79.9 (9.3)	—												
2. IAS Mainz 2 (*N* = 1509)	83.1 (10.5)	—	—											
3. BPQ-VSF (*N* = 484)	39.7 (8.7)	.44*** [.37, .51]	—	—										
4. PHQ-15 Mainz1 (*N* = 484)	23.6 (4.6)	-.15*** [-.24, -.07]	—	.08 [-.01, .17]	—									
5. PHQ-15 Mainz 2 (*N* = 1509)	21.9 (4.3)	—	-.14*** [-.19, -.09]	—	—	—								
6. PHQ-9 (*N* = 484)	15.4 (4.9)	-.19*** [-.27, -.10]	—	.04 [-.05, .13]	.66*** [.61, .71]	—	—							
7. MAIA-2 N (*N* = 484)	3.6 (0.6)	.49*** [.42, .55]	—	.43*** [.36, .50]	-.02 [-.11, .07]	—	-.09* [-.18, -.01]	—						
8. MAIA-2 ND (*N* = 484)	2.3 (0.7)	.15** [.06, .23]	—	.04 [-.05, .13]	-.12* [-.20, -.03]	—	-.12** [-.21, -.03]	.17*** [.09, .26]	—					
9. MAIA-2 NW (*N* = 484)	3.0 (0.7)	.10* [.01, .18]	—	-.04 [-.12, .05]	-.31*** [-.39, -.28]	—	-.33*** [-.49, -.25]	.06 [-.03, .15]	-.05 [-.14, .04]	—				
10. MAIA-2 AR (*N* = 484)	3.4 (0.7)	.41*** [.34, .48]	—	.28*** [.20, .36]	-.12** [-.20, -.03]	—	-.17*** [-.26, -.08]	.58*** [.52, .64]	.09* [.01, .18]	.26*** [.18, .34]	—			
11. MAIA-2 EA (*N* = 484)	3.8 (0.8)	.32*** [.24, .40]	—	.34*** [.26, .42]	.12* [.03, .20]	—	.06 [-.03, .14]	.55*** [.48, .61]	.10* [.01, .19]	.03 [-.06, .12]	.43*** [.35, .50]	—		
12. MAIA-2 SR (*N* = 484)	3.1 (0.9)	.30*** [.22, .38]	—	.18*** [.09, .27]	-.18*** [-.26, -.09]	—	-.25*** [-.33, -.16]	.37*** [.29, .44]	.12** [.04, .21]	.28*** [.19, .37]	.54*** [.48, .60]	.35*** [.27, .43]	—	
13. Maia-2 BL (*N* = 484)	3.2 (0.9)	.29 [.21, .37]	—	.25*** [.17, .34]	-.01 [-.09, .09]	—	-.10* [-.19, -.01]	.47*** [.40, .53]	.19*** [.10, .27]	.07 [-.02, .16]	.52*** [.45, .58]	.51*** [.44, .57]	.50*** [.43, .56]	—
14. Maia-2 Trust (*N* = 484)	3.7 (0.8)	.37*** [.29, .44]	—	.10* [.01, .19]	-.33*** [-.40, -.25]	—	-.46*** [-.53, -.39]	.36*** [.28, .43]	.17*** [.08, .26]	.23*** [.15, .31]	.43*** [.35, .50]	.23*** [.14, .31]	.50*** [.43, .56]	.45*** [.38, .52]
15. STAIT (*N* = 1509)	41.3 (10.8)	—	-.24*** [-.29, -.19)	—	—	.49*** [.45, .53]	—	—	—	—	—	—	—	—

Note. * p < .05, ** p < .01, *** p < .001 two-tailed, uncorrected; IAS = Interoceptive Accuracy Scale; BPQ-VSF = Body Perception Questionnaire Very Short Form; PHQ-15 = Patient Health Questionnaire 15-Item Version; PHQ-9 = Patient Health Questionnaire 9-Item Version; MAIA-2 = Multidimensional Assessment of Interoceptive Awareness Version 2 (Subscales: Noticing; Non-Distracting; Not-Worrying; Attention Regulation; Emotional Awareness; Self-Regulation; Body-Listening; Trusting); STAI-T = State-Trait Anxiety Inventory Trait Version.

Supplementary Table 3

Correlational analysis across questionnaires for the Potsdam and Vienna version of the IAS

Pearson's *r*	*M* (*SD*)	1 [95 % *CI*]	2 [95 % *CI*]	3 [95 % *CI*]	4 [95 % *CI*]	5 [95 % *CI*]	6 [95 % *CI*]	7 [95 % *CI*]	8 [95 % *CI*]	9 [95 % *CI*]	10 [95 % *CI*]
1. IAS Vienna	77.9	—									
(*N* = 642)	(10.3)										
2. IAS Potsdam	79.4	.70***	—								
(*N* = 808)	(11.0)	[.66, .74]									
3. TAS-20	48.7	-.29***	-.30***	—							
(*N* = 614)	(11.8)	[-.38, -.21]	[-.37, -.22]								
4. TAS-20 Desc	13.8	-.23***	-.24***	.86***	—						
(*N* = 614)	(4.5)	[-.31, -.14]	[-.32, -.17]	[.84, .88]							
5. TAS-20 Ident	17.3	-.30***	-.26***	.82***	.64***	—					
(*N* = 614)	(5.7)	[-.38, -.21]	[-.33, -.18]	[.80, .85]	[.59, .68]						
6. TAS-20 Ext	17.7	-.15**	-.20***	.67***	.41***	.23***	—				
(*N* = 614)	(4.7)	[-.24, -.05]	[-.28, -.12]	[.62, .71]	[.34, .47]	[.15, .30]					
7. BPQ Awa	81.8	.32***	.31***	-.08	-.04	-.01	-.15***	—			
(*N* = 808)	(18.7)	[.25, .39]	[.25, .37]	[-.16, .01]	[-.12, .04]	[-.08, .07]	[-.23, -.07]				
8. BPQ-Supra	23.1	-.13**	-.20***	.31***	.23***	.40***	.07	.17***	—		
(*N* = 808)	(6.9)	[-.20, -.05]	[-.27, -.14]	[.24, .38]	[.15, .30]	[.33, .46]	[-.01, .15]	[.10, .24]			
9. BPQ Supd	10.8	-.08*	-.10**	.17***	.11**	.27***	-.01	.22***	.54***	—	
(*N* = 808)	(3.9)	[-.16, -.01]	[-.17, -.03]	[.09, .24]	[.03, .19]	[.19, .34]	[-.09, .07]	[.15, .28]	[.49, .59]		
10. MAIA-2 N	3.9	.46***	.38***	-.13**	-.10**	-.17***	-.03	.28***	-.02	.07	—
(*N* = 614)	(0.9)	[.38, .53]	[.31, .44]	[-.21, -.05]	[-.18, -.02]	[-.24, -.09]	[-.10, .05]	[.20, .35]	[-.10, .06]	[-.01, .15]	
11. MAIA-2 ND	2.0	.10*	.09*	-.33***	-.32***	-.24***	-.22***	.06	-.01	-.05	.01
(*N* = 614)	(0.9)	[.01, .19]	[.01, .17]	[-.40, -.26]	[-.39, -.24]	[-.31, -.16]	[-.30, -.15]	[-.02, .14]	[-.08, .08]	[-.13, .03]	[-.07, .09]
12. MAIA-2 NW	2.6	.03	-.01	-.22***	-.14***	-.35***	.02	-.05	-.22***	-.16***	.08*
(*N* = 614)	(1.0)	[-.06, .12]	[-.08, .08]	[-.29, -.14]	[-.22, -.06]	[-.41, -.27]	[-.06, .10]	[-.13, .03]	[-.29, -.14]	[-.23, -.08]	[.01, .16]
13. MAIA-2 AR	3.2	.30***	.29***	-.31***	-.22***	-.36***	-.14***	.16***	-.21***	-.14***	.49***
(*N* = 614)	(0.9)	[.21, .38]	[.21, .36]	[-.38, -.24]	[-.29, -.14]	[-.43, -.29]	[-.22, -.06]	[.08, .23]	[-.28, -.13]	[-.21, -.06]	[.43, .55]
14. MAIA-2 EA	3.5	.34***	.31***	-.21***	-.15***	-.10*	-.26***	.27***	.04	.09*	.40***
(*N* = 614)	(1.0)	[.26, .42]	[.24, .38]	[-.28, -.13]	[-.22, -.07]	[-.18, -.02]	[-.34, -.19]	[.19, .34]	[-.04, .12]	[.02, .17]	[.33, .46]
15. MAIA-2 SR	2.7	.17***	.23***	-.34***	-.27***	-.33***	-.19***	.18***	-.12**	-.05	.34***
(*N* = 614)	(1.1)	[.08, .26]	[.15, .30]	[-.41, -.27]	[-.34, -.19]	[-.40, -.26]	[-.27, -.12]	[.10, .25]	[-.20, -.04]	[-.13, .03]	[.26, .40]
16. Maia-2 BL	3.1	.24***	.24***	-.18***	-.15***	-.12**	-.18***	.14***	-.02	.04	.52***
(*N* = 614)	(1.2)	[.15, .32]	[.16, .31]	[-.26, -.11]	[-.22, -.07]	[-.20, -.04]	[-.25, -.10]	[.06, .22]	[-.10, .06]	[-.04, .12]	[.46, .57]
17. Maia-2 Trust	3.8	.30***	.28***	-.40***	-.31***	-.45***	-.16***	.08	-.26***	-.21***	.34***
(*N* = 614)	(1.2)	[.21, .38]	[.21, .35]	[-.47, -.34]	[-.38, -.24]	[-.51, -.39]	[-.24, -.08]	[-.01, .16]	[-.34, -.19]	[-.29, -.13]	[.27, .41]
18. ICQ	49.2	-.50***	-.44***	.52***	.42***	.52***	.27***	-.16***	.33***	.23***	-.27***
(*N* = 614)	(8.6)	[-.57, -.43]	[-.50, -.37]	[.46, .58]	[.35, .48]	[.46, .58]	[.19, .34]	[-.24, -.09]	[.25, .40]	[.15, .30]	[-.34, -.20]
19. BDI	10.7	-.30*	-.27***	.50***	.39***	.53***	.23***	-.06	.38***	.27***	-.26***
(*N* = 226)	(8.7)	[-.51, -.05]	[-.39, -.15]	[.39, .59]	[.27, .49]	[.43, .62]	[.10, .35]	[-.19, .07]	[.26, .48]	[.15, .39]	[-.38, -.14]
20. ASI	24.7	-.06	-.15*	.40***	.29***	.46***	.16**	.06	.38***	.28***	-.14*
(*N* = 226)	(12.0)	[-.31, .20]	[-.27, -.02]	[.29, .51]	[.17, .41]	[.35, .56]	[.03, .29]	[-.08, .19]	[.27, .49]	[.15, .39]	[-.27, -.01]
21. STAIT	43.1	-.30*	-.25***	.53***	.44***	.56***	.21**	-.07	.36***	.25***	-.21**
(*N* = 226)	(11.4)	[-.51, -.05]	[-.36, -.12]	[.43, .62]	[.33, .54]	[.47, .65]	[.08, .33]	[-.20, .06]	[.24, .47]	[.12, .36]	[-.33, -.08]

Pearson's *r*	11<br>[95 % *CI*]	12<br>[95 % *CI*]	13<br>[95 % *CI*]	14<br>[95 % *CI*]	15<br>[95 % *CI*]	16<br>[95 % *CI*]	17<br>[95 % *CI*]	18<br>[95 % *CI*]	19<br>[95 % *CI*]	20<br>[95 % *CI*]
12. MAIA-2 NW	-.03	—								
(*N* = 614)	[-.11, .05]									
13. MAIA-2 AR	.10*	.24***	—							
(*N* = 614)	[.02, .18]	[.16, .31]								
14. MAIA-2 EA	.08*	-.06	.30***	—						
(*N* = 614)	[.01, .16]	[-.13, .02]	[.23, .37]							
15. MAIA-2 SR	.16***	.18***	.59***	.39***	—					
(*N* = 614)	[.08, .24]	[.10, .25]	[.54, .64]	[.32, .46]						
16. Maia-2 BL	.12**	.04	.45***	.42***	.54***	—				
(*N* = 614)	[.04, .19]	[-.04, .12]	[.39, .51]	[.36, .49]	[.48, .59]					
17. Maia-2 Trust	.14***	.24***	.50***	.22***	.56***	.46***	—			
(*N* = 614)	[.06, .21]	[.17, .31]	[.43, .55]	[.15, .30]	[.50, .61]	[.39, .52]				
18. ICQ	-.27***	-.06	-.33***	-.23***	-.22***	-.17***	-.33***	—		
(*N* = 614)	[-.34, -.20]	[-.14, .02]	[-.40, -.26]	[-.31, -.16]	[-.29, -.14]	[-.25, -.09]	[-.40, -.26]			
19. BDI	-.13*	-.38***	-.39***	-.07	-.43***	-.23***	-.59***	.40***	—	
(*N* = 226)	[-.26, -.01]	[-.48, -.26]	[-.49, -.27]	[-.20, .06]	[-.53, -.31]	[-.35, -.11]	[-.67, -.50]	[.28, .50]		
20. ASI	-.12	-.59***	-.35***	-.05	-.33***	-.09	-.43***	.31***	.46***	—
(*N* = 226)	[-.25, .01]	[-.67, -.50]	[-.46, -.23]	[-.17, .09]	[-.45, -.21]	[-.22, .04]	[-.53, -.31]	[.19, .42]	[.36, .56]	
21. STAIT	-.14*	-.46***	-.40***	-.07	-.51***	-.27***	-.63***	.44***	.79***	.57***
(*N* = 226)	[-.26, -.01]	[-.56, -.35]	[-.51, -.29]	[-.20, .06]	[-.60, -.40]	[-.39, -.15]	[-.70, -.55]	[.33, .54]	[.73, .83]	[.47, .65]

Note. * $p < .05$, ** $p < .01$, *** $p < .001$ two-tailed, uncorrected; IAS = Interoceptive Accuracy Scale; TAS-20 = Toronto Alexithymia Scale 20-Items (Subscales: Difficulty Describing Feelings; Difficulty Identifying Feelings; Externally oriented thinking); BPQ = Body Perception Questionnaire (Subscales: General Body Awareness, Supradiaphragmatic, Subdiaphragmatic); MAIA-2 = Multidimensional Assessment of Interoceptive Awareness Version 2 (Subscales: Noticing; Non-Distracting; Not-Worrying; Attention Regulation; Emotional Awareness; Self-Regulation; Body-Listening; Trusting); ICQ = Interoceptive Confusion Questionnaire; BDI = Beck Depression Inventory; ASI = Anxiety Sensitivity Index; STAI-T = State-Trait Anxiety Inventory Trait Version.

Supplementary Table 4

Correlational analysis across questionnaires for the Giessen version of the IAS

Pearson's *r*	*M* (*SD*)	1 [95 % *CI*]	2 [95 % *CI*]	3 [95 % *CI*]	4 [95 % *CI*]	5 [95 % *CI*]	6 [95 % *CI*]	7 [95 % *CI*]	8 [95 % *CI*]	9 [95 % *CI*]	10 [95 % *CI*]	11 [95 % *CI*]	12 [95 % *CI*]	13 [95 % *CI*]
1. IAS	87.1	—												
(*N* = 522)	(9.5)													
2. FFMQ Obs	27.3	.27***	—											
(*N* = 522)	(5.0)	[.19, .35]												
3. FFMQ Des	27.3	.31***	.23***	—										
(*N* = 522)	(6.5)	[.23, .39]	[.15, .31]											
4. NEO-FFI N	17.0	-.19***	-.06	-.33***	—									
(*N* = 522)	(5.3)	[-.27, -.11]	[-.14, .03]	[-.41, -.25]										
5. MAIA-2 N	4.2	.46***	.41***	.29***	-.11*	—								
(*N* = 522)	(0.8)	[.39, .53]	[.34, .48]	[.21, .37]	[-.20, -.03]									
6. MAIA-2 ND	3.2	.06	-.01	.16***	-.16***	.02	—							
(*N* = 522)	(0.9)	[-.03, .15]	[-.10, .07]	[.08, .24]	[-.24, -.07]	[-.07, .10]								
7. MAIA-2 NW	3.5	.08	-.04	.13**	-.37***	.02	-.07	—						
(*N* = 522)	(0.9)	[-.01, .16]	[-.12, .05]	[.04, .21]	[-.44, -.29]	[-.06, .11]	[-.16, .01]							
8. MAIA-2 AR	3.7	.38***	.36***	.42***	-.36***	.50***	.01	.29***	—					
(*N* = 522)	(0.9)	[.31, .45]	[.29, .43]	[.34, .49]	[-.43, -.28]	[.43, .56]	[-.08, .09]	[.21, .37]						
9. MAIA-2 EA	4.4	.32***	.41***	.23***	-.05	.46***	.03	-.04	.39***	—				
(*N* = 522)	(0.9)	[.24, .40]	[.34, .48]	[.15, .31]	[-.14, .03]	[.39, .52]	[-.06, .12]	[-.13, .05]	[.32, .46]					
10. MAIA-2 SR	3.3	.25***	.28***	.28***	-.41***	.37***	.08	.21***	.54***	.40***	—			
(*N* = 522)	(1.1)	[.17, .33]	[.20, .36]	[.20, .36]	[-.48, -.34]	[.29, .44]	[-.01, .16]	[.13, .29]	[.47, .60]	[.33, .47]				
11. Maia-2 BL	3.2	.25***	.34***	.35***	-.28***	.42***	.17***	-.01	.43***	.47***	.56***	—		
(*N* = 522)	(1.1)	[.17, .33]	[.27, .42]	[.27, .42]	[-.35, -.19]	[.34, .48]	[.08, .25]	[-.10, .08]	[.35, .49]	[.40, .53]	[.50, .62]			
12. Maia-2 Trust	4.3	.31***	.25***	.35***	-.50***	.31***	.14**	.22***	.47***	.27***	.55***	.49***	—	
(*N* = 522)	(1.3)	[.23, .39]	[.16, .33]	[.27, .42]	[-.56, -.43]	[.23, .39]	[.06, .23]	[.14, .30]	[.40, .53]	[.19, .35]	[.49, .61]	[.42, .55]		
13. STAIT	44.7	-.27***	-.06	-.32***	.81***	-.13**	-.17***	-.40***	-.37***	-.12**	-.47***	-.32***	-.57***	—
(*N* = 522)	(10.9)	[-.34, -.17]	[-.14, .03]	[-.39, -.24]	[.77, .83]	[-.21, -.04]	[-.25, -.08]	[-.47, -.32]	[-.44, -.29]	[-.20, -.03]	[-.53, -.40]	[-.39, -.24]	[-.62, -.51]	
14. ADS	39.4	-.21***	-.06	-.29***	.71***	-.08	-.16***	-.31***	-.30***	-.08	-.41***	-.25***	-.51***	.80***
(*N* = 522)	(11.1)	[-.29, -.12]	[-.15, .03]	[-.37, -.21]	[.66, .75]	[-.17, .01]	[-.24, -.07]	[-.38, -.23]	[-.37, -.22]	[-.17, .01]	[-.48, -.34]	[-.32, -.16]	[-.57, -.44]	[.76, .83]

Note. * $p < .05$, ** $p < .01$, *** $p < .001$ two-tailed, uncorrected; IAS = Interoceptive Accuracy Scale; FFMQ = Five Facet Mindfulness Questionnaire (Subscales: Observation; Description); MAIA-2 = Multidimensional Assessment of Interoceptive Awareness Version 2 (Subscales: Noticing; Non-Distracting; Not-Worrying; Attention Regulation; Emotional Awareness; Self-Regulation; Body-Listening; Trusting); STAI-T = State-Trait Anxiety Inventory Trait Version; ADS = German Version of the Center for Epidemiologic Studies Depression Scale.

Supplementary Notes 6: Comparison of convergent validity across IAS versions.

Multidimensional Assessment of Interoceptive Awareness, Version 2.

We found no evidence that the relation between the IAS and the MAIA-2 subscales differ between the versions for the major part. From the 48 possible comparisons, only two reached significance.

Supplementary Table 5

Comparison of correlations between the IAS and MAIA-2 across IAS versions.

	Mainz vs. Vienna	Mainz vs. Potsdam	Mainz vs. Giessen	Vienna vs. Potsdam	Vienna vs. Giessen	Potsdam vs. Giessen
1. MAIA-2 Noticing	-0.42	0.52	0.53	0.91	0.93	-0.03
2. MAIA-2 Not Distracting	0.81	0.96	1.34	0.14	0.43	0.28
3. MAIA-2 Not Worrying	1.11	**1.99***	0.31	0.83	-0.81	-1.7
4. MAIA-2 Attention Regulation	1.89	1.89	0.58	0	-1.3	-1.39
5. MAIA-2 Emotional Awareness	-0.31	-0.64	0	-0.31	0.31	0.32
6. MAIA-2 Self-regulation	**2.01***	0.95	0.88	-1.01	-1.21	-1.21
7. MAIA-2 Body Listening	0.78	0.32	0.66	-.045	-0.17	-0.17
8. MAIA-2 Trust	1.10	1.26	1.01	0.15	-0.16	-0.16

Note. Fischer's Z values for the comparison of the different versions of the IAS with the MAIA-2. * indicates two-tailed $p < .05$

Interoceptive Confusion Questionnaire

Potsdam and Vienna versions were compared with the ICQ, revealing no evidence for significant differences: Fischer's $Z = -0.31$, $p = .754$

Body Perception Questionnaire Very Short Form

BPQ-VSF. Mainz, Potsdam, and Vienna versions were compared with the BPQ-VSF. Results suggested that the Potsdam and Vienna versions showed a similar relationship with

the BPQ-VSF (Potsdam vs. Vienna: Fischer's $Z = 0.36$, $p = .712$). However, differences emerged with the Mainz version (Mainz vs. Potsdam: Fischer's $Z = 3.88$, $p < .001$, Mainz vs. Vienna: Fischer's $Z = 3.53$, $p < .001$). It should be noted that, unlike the Mainz sample that was administered the BPQ-VSF, the Vienna and Potsdam samples underwent the longer BPQ-SF from which the BPQ-VSF could be extracted. Although across samples and versions, the relation between the BPQ-VSF and the IAS was positive, the size of the relationship might have been influenced by the administered format of the BPQ.

Toronto Alexithymia Scale-20

Potsdam and Vienna versions were compared with the TAS-20 total scores. Results indicated that both versions of the IAS showed a comparable relationship with TAS-20 total scores (Fischer's $Z = 0.12$, $p = .897$).

State-Trait Anxiety Inventory – Trait Version

Mainz, Potsdam, and Giessen versions were compared with the STAI-T. IAS versions showed a similar relationship with the STAI-T scores (Mainz vs. Potsdam: Fischer's $Z = 0.26$, $p = .780$, Mainz vs. Giessen: Fischer's $Z = -0.13$, $p = .895$, Giessen vs. Potsdam: Fischer's $Z = 0.08$, $p = .936$).

Supplementary Notes 7: Correlations between the IAS Potsdam and HCT

Some participants from the Potsdam samples completed the IAS Potsdam and the HCT (N = 41). Overall, we did not find evidence for any significant relationship between the IAS Potsdam version and the HCT. As we found a significant relationship between Interoceptive Sensibility ratings and the IAS Vienna version (r = .21, p = .034) but not the Potsdam version (r = -.01, p = .934), we conducted an equivalence test[1] with r = .21 as the lower and upper bound. The equivalence test indicated that both the null-hypothesis test (p =.936) and equivalence test (p = .121) were nonsignificant. This means that the correlation of the IAS Potsdam with the HCT sensibility ratings is undetermined. Thus, we can conclude that the observed correlation is not statistically different from 0 nor statistically equivalent to r = .21.

Supplementary Table 6

Correlations between the IAS Potsdam and HCT

Variable	*M*	*SD*	*1*	*2*	*3*
1. IAS	81.2	11.6			
2. HCT accuracy	2.6	1.3	-.02		
3. HCT Interoceptive Sensibility	60.9	18.5	-.01	-.23	
4. HCT Interoceptive Awareness	39.8	24.9	.07	-.82***	.65***

Note. * indicates p < .05, ** indicates p < .01, *** indicates p < .001.

Supplementary Notes 8

Supplementary Figure 2

Parallel Analysis of the Mainz Version

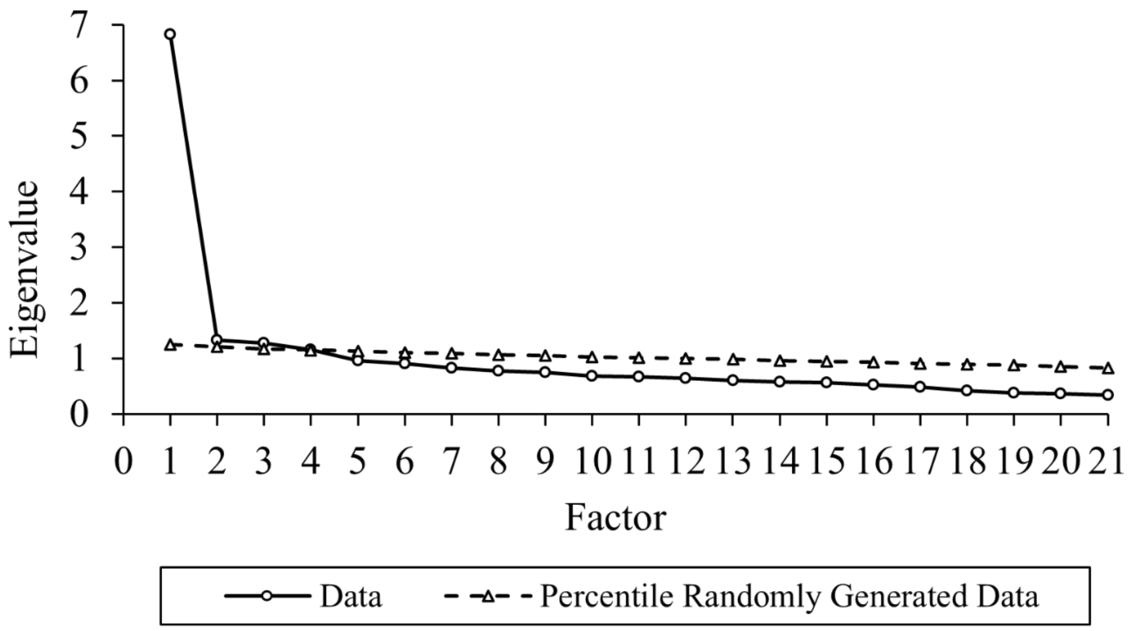

Supplementary Notes 9: Additional analysis of Vienna and Potsdam samples and versions

Following our preregistration (aspredicted.org/e6tr3.pdf), for our main analysis we pooled together the Vienna and Potsdam samples to increase the sample size available for the Vienna and Potsdam translations. *Despite our pre-planned analysis strategy, we assessed whether samples differed in age, gender and IAS scores.* We observed that the Vienna and Potsdam samples differed regarding both age, $t(572.41) = -11.46$, $p < .001$, and gender, $\chi^2(2) = 62.19$, $p < .001$, however, mean scores of the IAS Vienna, $t(567.15) = .59$, $p = .56$, and Potsdam, $t(805.96) = -.47$, $p = .64$, versions were not different for the Vienna and Potsdam samples.

Additionally, we computed correlational analysis for the questionnaires available in both the Vienna and Potsdam samples (MAIA 2, BPQ, ICQ, TAS-20). We divided the IAS into samples and versions meaning that we compared 4 versions of the IAS (Vienna sample & Vienna version, Vienna sample & Potsdam version, Potsdam sample & Vienna version, Potsdam sample & Potsdam version). A forestplot with all correlations is depicted in Supplementary Figure 3.

Supplementary Figure 3

Forestplot comparing Vienna and Potsdam samples and versions (TAS-20, BPQ, and ICQ)

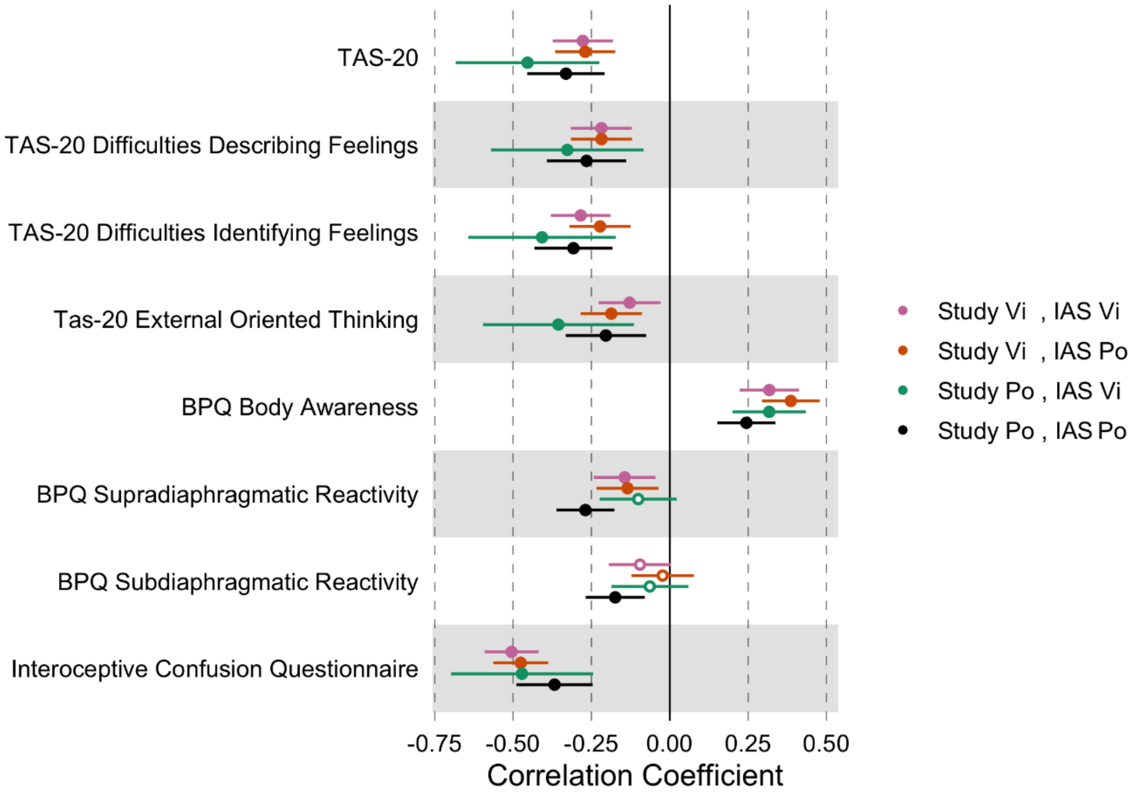

Note. Po = Potsdam; Vi = Vienna; filled circles indicate two-tailed, Bonferroni-corrected significant correlations at $p < .003$; Error bars represent 95 % CIs of Pearson correlation coefficients.

Supplementary Figure 4

Forestplot comparing Vienna and Potsdam samples and versions (MAIA)

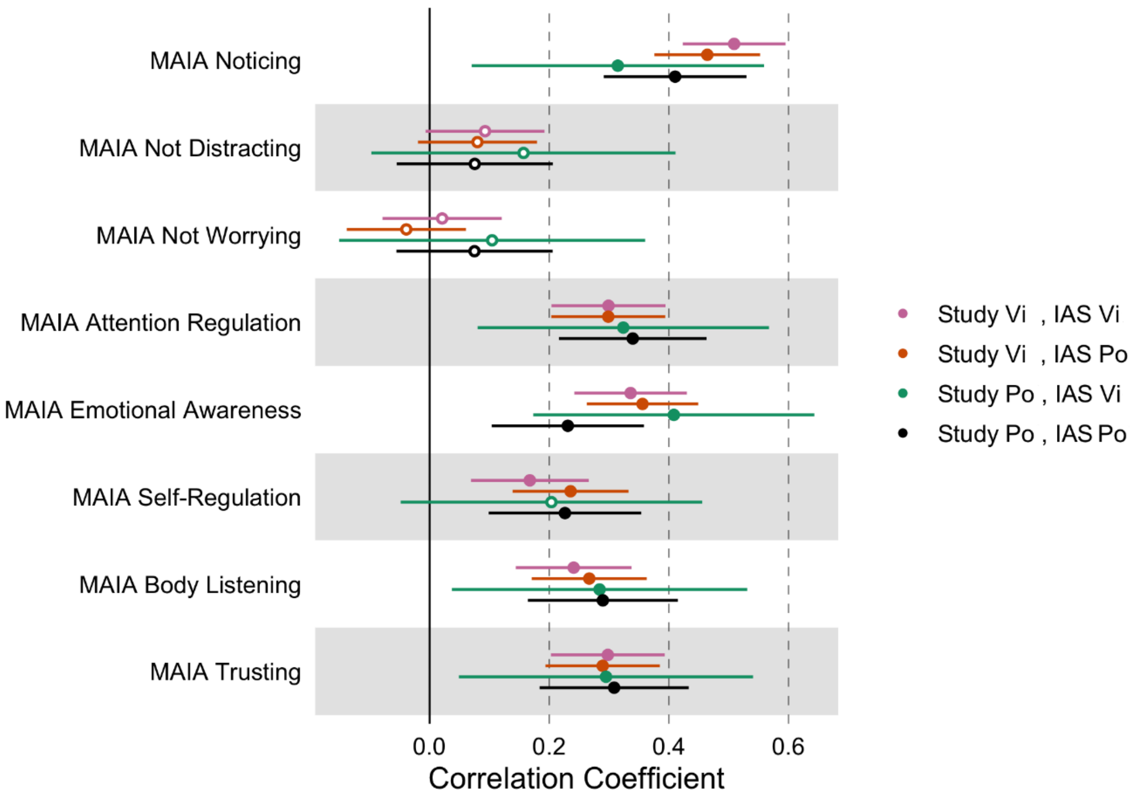

Note. Po = Potsdam; Vi = Vienna; filled circles indicate two-tailed, Bonferroni-corrected significant correlations at $p < .003$; Error bars represent 95 % *CI*s of Pearson correlation coefficients.

Supplementary Notes 10: Explorative Analysis of Objective Interoception Scores

In our main analysis, no evidence for a significant relationship between IAS and objective indices of interoception (HCT- and HDT-scores) was found. Following up on these results we conducted several regression analyses to control for the impact of potential confounding variables (gender, age, BMI, or time-counting abilities). For the HCT data results can be found in Supplementary Table 7. For the HDT data results can be found in Supplementary Table 8. Further, for the HDT we used percentage correct responses as an index for objective interoceptive accuracy in our main analysis. However, in the literature, some authors also use d' as an index for objective interoceptive accuracy[2]. Thus, we calculated d' using the *psych* r-package[3] and ran the same analysis (see Supplementary Table 9).

The IAS measures a range of interoceptive sensations, while the objective interoceptive measurements used here only focus on heartbeat perception. As the perception of different interoceptive modalities is not necessarily related[4] the missing evidence for significant relationships regarding our objective interoceptive indices and the IAS may be caused by the breadth of the interoceptive signals covered by the scales. Therefore, in an exploratory analysis, we correlated HDT and HCT scores with the IAS items measuring the perception of heartbeat and respiration. However, for both items also no evidence for a significant correlation to HCT scores (heartbeat: $r = .06$; $p = .51$, respiration: $r = .11$, $p = .27$) or HDT scores (heartbeat: $r =-.02$, $p = .84$; respiration: $r = -,09$, $p = .44$) was found.

Supplementary Table 7

Explorative Analysis for IAS and HCT

Term	*β*	*SE*	*t*	*p*
(Intercept)	67.68	7.67	8.82	< .001
HCT_logAcc	0.55	0.72	0.77	.443
gender	1.45	1.85	0.78	.436
age	-0.23	0.14	-1.71	.091
BMI	0.33	0.33	1.02	.311
TCT_Acc	0.05	0.06	0.93	.363

Note. Regression analysis for the impact of log-transformed HCT scores on IAS, controlling for gender, age, BMI, and time counting.

Supplementary Table 8

Explorative Analysis for IAS and HDT

Term	*β*	*SE*	*t*	*p*
(Intercept)	70.46	8.06	8.74	< .001
hdt_perc_correct	7.54	7.26	1.04	.303
gender	0.31	2.13	0.15	.883
age	-0.32	0.16	-2.05	.044
BMI	0.49	0.39	1.25	.214

Note. Regression analysis for the impact of HDT scores on IAS, controlling for gender, age, and BMI.

Supplementary Table 9

Explorative Analysis for IAS and HDT (dprime)

Term	*β*	*SE*	*t*	*p*
(Intercept)	73.87	7.76	9.52	< .001
dprime	0.75	1.29	0.58	.562
gender	0.38	2.14	0.18	.858
age	-0.33	0.16	-2.05	.044
BMI	0.50	0.40	1.26	.213

Note. Regression analysis for the impact of HDT dprime scores on IAS, controlling for gender, age, and BMI.

Supplementary References

1. Lakens, D., Scheel, A. M. & Isager, P. M. Equivalence Testing for Psychological Research: A Tutorial. *Advances in Methods and Practices in Psychological Science* **1,** 259–269; 10.1177/2515245918770963 (2018).

2. Brener, J. & Ring, C. Towards a psychophysics of interoceptive processes: the measurement of heartbeat detection. *Philosophical transactions of the Royal Society of London. Series B, Biological sciences* **371**; 10.1098/rstb.2016.0015 (2016).

3. Revelle, W. *Psych. Procedures for Psychological, Psychometric, and Personality Research* (2020).

4. Garfinkel, S. N. *et al.* Interoceptive dimensions across cardiac and respiratory axes. *Philosophical transactions of the Royal Society of London. Series B, Biological sciences* **371**; 10.1098/rstb.2016.0014 (2016).
