# Supplementary material for: A multi-site German validation of the Interoceptive Accuracy Scale and its relation to psychopathological symptom burden
Source: Commun Psychol. 2023 Sep 5;1:14. doi: 10.1038/s44271-023-00016-x (PMC11332230; doi:10.1038/s44271-023-00016-x)
Supplement: Supplementary file 1 [file 44271_2023_16_MOESM3_ESM.pdf]

nature portfolio

Corresponding author(s): Sebastian Brand, Johannes Gutenberg-University Mainz, Germany sebbrand@uni-mainz.de

Last updated by author(s): Jul 26, 2023

Reporting Summary

Nature Portfolio wishes to improve the reproducibility of the work that we publish. This form provides structure for consistency and transparency in reporting. For further information on Nature Portfolio policies, see our Editorial Policies and the Editorial Policy Checklist.

Statistics

For all statistical analyses, confirm that the following items are present in the figure legend, table legend, main text, or Methods section.

n/a	Confirmed	
☐	☒	The exact sample size (*n*) for each experimental group/condition, given as a discrete number and unit of measurement
☐	☒	A statement on whether measurements were taken from distinct samples or whether the same sample was measured repeatedly
☐	☒	The statistical test(s) used AND whether they are one- or two-sided<br>*Only common tests should be described solely by name; describe more complex techniques in the Methods section.*
☐	☒	A description of all covariates tested
☐	☒	A description of any assumptions or corrections, such as tests of normality and adjustment for multiple comparisons
☐	☒	A full description of the statistical parameters including central tendency (e.g. means) or other basic estimates (e.g. regression coefficient) AND variation (e.g. standard deviation) or associated estimates of uncertainty (e.g. confidence intervals)
☐	☒	For null hypothesis testing, the test statistic (e.g. *F*, *t*, *r*) with confidence intervals, effect sizes, degrees of freedom and *P* value noted<br>*Give P values as exact values whenever suitable.*
☒	☐	For Bayesian analysis, information on the choice of priors and Markov chain Monte Carlo settings
☒	☐	For hierarchical and complex designs, identification of the appropriate level for tests and full reporting of outcomes
☐	☒	Estimates of effect sizes (e.g. Cohen's *d*, Pearson's *r*), indicating how they were calculated

Our web collection on statistics for biologists contains articles on many of the points above.

Software and code

Policy information about availability of computer code

Data collection	Applications for creating (online) questionnaires (e.g., SoSciSurvey, Unipark, Sona Systems) were used for data collection. In addition, a two-lead setup with an MP-160 BIOPAC system and a single-lead setup with an ADInstruments Powerlab 4/35 and Bioamp were used for ECG.
Data analysis	The software IBM SPSS Statistics (IBM Corp., 2020), Mplus (Muthén & Muthén, 1998), and R version 4.0.5 (R Core Team, 2013) were used to perform the statistical analyses. Within R, we used the packages tidyverse, psych, lavaan, lme4, and cocor.

For manuscripts utilizing custom algorithms or software that are central to the research but not yet described in published literature, software must be made available to editors and reviewers. We strongly encourage code deposition in a community repository (e.g. GitHub). See the Nature Portfolio guidelines for submitting code & software for further information.

Data

Policy information about availability of data

All manuscripts must include a data availability statement. This statement should provide the following information, where applicable:

- Accession codes, unique identifiers, or web links for publicly available datasets
- A description of any restrictions on data availability
- For clinical datasets or third party data, please ensure that the statement adheres to our policy

The dataset for the study is available at Open Science Framework. The DOI for this website is: https://doi.org/ 10.17605/OSF.IO/3F2H6. The permanent URL pointing to this raw data is: https://osf.io/3f2h6/.

Human research participants

Policy information about studies involving human research participants and Sex and Gender in Research.

Reporting on sex and gender	Data on gender were collected on the basis of self-report (forced-choice with an additional open response). It was analyzed whether the sumscores of the Interoceptive Accuracy Scale (IAS) differed between genders. Results refer to gender only, as no methods were used to define sex.
Population characteristics	Age and gender of participants were assessed to descibe population characteristics
Recruitment	Participants were recruited through universitiy email lists (i.e., university students), social media (i.e., interested volunteers), and online platforms (e.g., prolific.co). Therefore, the samples can be considered heterogeneous in terms of selection bias.
Ethics oversight	Data collection was approved by the Ethic Committee of the Psychological Institutes of JGU Mainz, University of Vienna and Potsdam, and the JLU Giessen

Note that full information on the approval of the study protocol must also be provided in the manuscript.

Field-specific reporting

Please select the one below that is the best fit for your research. If you are not sure, read the appropriate sections before making your selection.

☐ Life sciences ☒ Behavioural & social sciences ☐ Ecological, evolutionary & environmental sciences

For a reference copy of the document with all sections, see nature.com/documents/nr-reporting-summary-flat.pdf

Behavioural & social sciences study design

All studies must disclose on these points even when the disclosure is negative.

Study description	Quantitative data were collected to validate the German Version of the Ineroceptive Accuracy Scale. In addition, physiological data were collected (Heartbeat Counting Task) to operationalize cardiac interoceptive accuracy.
Research sample	The present study is a multi-site study comprising seven samples. The samples from Mainz were recruited from the German general population. The Vienna samples composed of Austrian students and people from the German-speaking general population. The Potsdam and Giessen samples consisted mainly of German university students (in Giessen also from the German general population). Demographic information is provided in the methods section of the manuscript for each sample
Sampling strategy	Individual sample sizes were initially chosen to be consistent with previous validation studies (e.g., Murphy et al., 2020 or Campos et al., 2022) and common rules of thumb (e.g., 10 cases per variable for exploratory analysis), except for the second Mainz sample, which was chosen based on power analysis to meet the requirements for the additional COVID-related questions (see Jungmann & Witthöft, 2020)
Data collection	Data were collected mainly online, with participants completing questionnaires at home without supervision (using the applications described above). Some participants in Vienna and Potsdam additionally performed a heartbeat counting task in each university laboratory under the supervision of trained researchers
Timing	Individual data collection times were: Mainz Sample 1 (Winter 2019) and Sample 2 (Spring 2020), Vienna Sample 1 (Winter 2021) and Sample 2 (Spring 2021 to Spring 2022), Potsdam Sample 1 (Summer 2020 to Winter 2021) and Sample 2 (Spring 2021 and Winter 2022), and Giessen (Fall 2021)
Data exclusions	Participants were excluded if they did not report a high proficiency German level, were underaged, left items unanswered, and/or responded to fast or slow. Overall, about 4.8% of participants had to be excluded after data collection.
Non-participation	No participants dropped out / declined participation
Randomization	Participants were not allocated into experimental groups

Reporting for specific materials, systems and methods

We require information from authors about some types of materials, experimental systems and methods used in many studies. Here, indicate whether each material, system or method listed is relevant to your study. If you are not sure if a list item applies to your research, read the appropriate section before selecting a response.

Materials & experimental systems

n/a	Involved in the study	
☒	☐	Antibodies
☒	☐	Eukaryotic cell lines
☒	☐	Palaeontology and archaeology
☒	☐	Animals and other organisms
☒	☐	Clinical data
☒	☐	Dual use research of concern

Methods

n/a	Involved in the study	
☒	☐	ChIP-seq
☒	☐	Flow cytometry
☒	☐	MRI-based neuroimaging
